# Peripheral Brain-Derived Neurotrophic Factor and Contactin-1 Levels in Patients with Attention-Deficit/Hyperactivity Disorder

**DOI:** 10.3390/jcm8091366

**Published:** 2019-09-02

**Authors:** Liang-Jen Wang, Chih-Ching Wu, Min-Jing Lee, Miao-Chun Chou, Sheng-Yu Lee, Wen-Jiun Chou

**Affiliations:** 1 Department of Child and Adolescent Psychiatry, Kaohsiung Chang Gung Memorial Hospital and Chang Gung University College of Medicine, Kaohsiung 83301, Taiwan; 2 Department of Otolaryngology-Head & Neck Surgery, Linkuo Chang Gung Memorial Hospital, Taoyuan 33302, Taiwan; 3 Department of Medical Biotechnology and Laboratory Science, Chang Gung University, Tao-Yuan 33302, Taiwan; 4 Department of Psychiatry, Kaohsiung Veterans General Hospital, Kaohsiung 83301, Taiwan; 5 Department of Psychiatry, School of Medicine, and Graduate Institute of Medicine, College of Medicine, Kaohsiung Medical University, Kaohsiung 83301, Taiwan

**Keywords:** ADHD, BDNF, CNTN1, proteinomics, biomarkers, neuropsychological test

## Abstract

Brain-derived neurotrophic factor (BDNF) facilitates neuronal growth and plasticity, and is crucial for learning and memory. Contactin-1 (CNTN1) is a member of the subfamily of neural immunoglobulin and is involved in the formation of axon connections in the developing nervous system. This cross-sectional study investigates whether BDNF and CNTN1 affect susceptibility to attention deficit/hyperactivity disorder (ADHD). A total of 136 drug-naïve patients with ADHD (108 boys and 28 girls) and 71 healthy controls (45 boys and 26 girls) were recruited. Blood samples were obtained to measure the plasma levels of BDNF and CNTN1 in each child. We found that BDNF levels in the ADHD boys exceeded those in the control boys, but BDNF levels in the ADHD girls were lower than those in the control girls. Boys who had higher BDNF levels performed worse on the Wechsler Intelligence Scale for Children—Fourth Edition, but girls who had higher BDNF levels made fewer omission errors in the Conners’ Continuous Performance Test. However, CNTN1 level did not differ significantly between patients and controls, and were not correlated to ADHD characteristics, regardless of gender. The findings suggest BDNF may influence sex-specific susceptibility to ADHD, but CNTN1 was not associated with ADHD pathophysiology.

## 1. Introduction

Attention deficit/hyperactivity disorder (ADHD) is characterized by inattention, impulsivity, and hyperactivity [1]. It is now recognized as the most common neurodevelopmental disorder in childhood [2], and the prevalence of ADHD among school-age children may be as high as 5–10% [3]. The identification of biomarkers for ADHD may assist in objective diagnosis, the monitoring of response to treatment and the prediction of outcomes [4]. Neurotransmitters in the noradrenergic system or neurotrophin substrates have been proposed as potential biomarkers of ADHD [5]. Notably, sex differences in clinical characteristics have been well established [6], but whether biomarkers have sex-specific effects on susceptibility to ADHD remains unclear.

Brain-derived neurotrophic factor (BDNF) has an important role in neuronal survival and growth, serving a neurotransmitter modulator [7]. BDNF is an important neurotrophin in the central nervous system, and is associated with neuronal plasticity, which is critical for learning and memory [8]. Higher peripheral levels of BDNF have been linked to fewer behavioral problems and clinical symptoms in preschool children with neuro-developmental disorders [9]. Both animal models and human studies suggest that BDNF Val66Met genotype is associated with ADHD [10]. Studies suggest that dysfunctional BDNF is a possible contributor to the pathology and symptoms of ADHD [11]. Nonetheless, the findings about whether peripheral levels of BDNF can serve as reliable ADHD biomarkers were inconsistent among studies [12,13,14,15,16]. One possible explanation for this inconsistency is the moderating effect that gender has on the relationship between BDNF and ADHD. An animal study has established that only male rats, but not female ones, exhibit falling BDNF expression in the postnatal cerebellum [17]. A recent meta-analysis has demonstrated that BDNF levels are significantly higher in males with ADHD than in male controls [14], but the same difference was not observed in girls. Sex-related hormonal, genetic, and epigenetic factors interact with BDNF signaling at various levels and in complex ways between genders [18]. Therefore, we supposed that the relationship between BDNF levels and ADHD characteristics may differ between boys and girls.

Contactin-1, also known as CNTN1, is a protein that is encoded by the CNTN1 gene. The CNTN1 gene is a member of the subfamily of neural immunoglobulin (Ig) and is involved in the formation of axon connections in the developing nervous system [19]. CNTN1 is a novel adhesion protein involved in neuronal migration that regulates the process formation of newborn cortical neurons [20]. BDNF also has a role in regulating synaptic functions, by enhancing neuronal excitability through GABAergic neurons [21]. Therefore, CNTN1 and BDNF both facilitate neuron axons guidance [22], and may play a role in the pathophysiology of ADHD. Other members of the Ig subfamily, contactin-4, -5, and -6 (CNTN4, -5, and -6), participate in pathways that are important for brain development [23]. Recent genetic studies of neuropsychiatric disorders have identified CNTN4, -5, and -6 as candidate genes in neurodevelopmental disorders, including autism spectrum disorders (ASD) and ADHD [24]. However, whether peripheral levels of CNTN1 are associated with ADHD had yet not been examined.

We hypothesize that BDNF and CNTN1 are involved in ADHD susceptibility, and patients with ADHD may exhibit lower BDNF/CNTN1 levels than control subjects. Furthermore, we have reasoned that the relationship of BDNF/CNTN1 levels and ADHD characteristics may differ between boys and girls. To elucidate the role of BDNF and CNTN1 in susceptibility to ADHD, a cross-sectional study was carried out to examine whether levels of BDNF and CNTN1 differ between ADHD patients and healthy controls. Additionally, whether the relationships among BDNF, CNTN1, and ADHD characteristics differ between genders.

## 2. Material and Methods

### 2.1. Study Participants

The Institutional Review Board at Chang Gung Hospital in Taiwan approved the research protocol. Patients with ADHD being treated in the outpatient Department of Child Psychiatry at Chang Gung Children’s Hospital in Taiwan were recruited for this cross-sectional study and informed consent was obtained in writing from all participants or their guardians. The inclusion criteria were as follows; (a) clinical diagnosis of ADHD by a senior child psychiatrist based on the DSM-IV-TR through structured interviews using the Chinese epidemiologic version of the Schedule for Affective Disorders and Schizophrenia for School-Age Children (K-SADS-E) [25]; (b) between the ages of six and 16 years old; and (c) a drug-naïve patient or a patient with a diagnosis who had not taken an ADHD medication for at least six months. Any patient with a history of comorbid autism spectrum disorder, intellectual disability, major depressive disorder, bipolar disorder, psychosis, epilepsy, or brain injury was excluded.

The healthy control subjects were recruited from the school in the same catchment areas through advertising or were the healthy classmates of ADHD patients. The control group included children without ADHD or other psychiatric disorders (such as intellectual disabilities, autism spectrum disorder, bipolar disorders, major depressive disorders, psychotic disorders, substance dependence, epilepsy, or severe head trauma) from the same catchment area as the non-control patients to serve as healthy controls. Overall, a total of 136 patients with ADHD (108 boys and 28 girls) and 71 healthy controls (45 boys and 26 girls) were recruited for this investigation.

### 2.2. Laboratory Testing to Determine BDNF and CNTN1 Levels

Blood samples were collected at 8:00 a.m. from participants who had fasted overnight. The BDNF and CNTN1 levels in plasma samples were obtained using the Human Magnetic Luminex Assay (LXSAHM; R&D Systems, Minneapolis, MN, USA). The assays were conducted according to the protocol that was provided by R&D Systems. Briefly, a 96-well filter-bottom microplate (Millipore, Billerica, MA, USA) was blocked for 10 min with assay buffer (R&D Systems). To generate standard curves, four-fold serial dilutions of standards were prepared in serum diluent (R&D Systems). 50 μL of standards and plasma samples (1:2 dilution) were added to the wells, which contained 50 μL of the immunobead mixture. The microplate was incubated for 40 min at room temperature in the dark and then washed three times with washing buffer (R&D Systems) using a vacuum manifold. A mixture of biotin-conjugated secondary antibodies (R&D Systems) was added. After 40 min of incubation and three washes, streptavidin-phycoerythrin (R&D Systems) was added. After 10 min, the immunobeads were washed three times, resuspended in 50 μL assay buffer, and analyzed using the Bio-Plex 200 system (Bio-Rad Laboratories, Hercules, CA, USA).

### 2.3. Clinical Measurements

A senior psychiatrist interviewed the ADHD patients and healthy control subjects using the K-SADS-E diagnostic tool. An experienced child psychologist conducted a neurocognitive assessment using the Wechsler Intelligence Scale for Children–Fourth Edition (WISC-IV). The Swanson, Nolan, and Pelham Version IV Scale (SNAP-IV) parent form and SNAP-IV teacher form were completed by each patient’s parents and teacher of both ADHD patients and controls, respectively.

The WISC-IV is an individually administered and norm-referenced tool for determining the intelligence of children from 6 to 16 years old [26]. The WISC-IV comprises ten core and five supplemental subtests. The core subtests address the following four factor indices; Verbal Comprehension Index (VCI), Perceptual Reasoning Index (PRI), Working Memory Index (WMI), and Processing Speed Index (PSI). The Full-Scale Intelligence Quotient (FSIQ) is obtained using the 10 core subtests. Both the factor indices and the FSIQ have a population mean of 100 and a standard deviation of 15 [27].

The SNAP-IV is a 26-item questionnaire that is completed by parents or teachers and used to evaluate ADHD symptoms and severity [28]. The 26 items comprise 18 that are related to ADHD symptoms (nine concerning inattention and nine concerning hyperactivity/impulsivity) and eight for oppositional defiant disorder (ODD) symptoms, as defined by the DSM-IV. Each item is scored from zero to three on a Likert scale.

The CPT is a 14-minute computerized test that primarily assesses attention and impulse control [29]. Briefly, participants are required to respond to stimuli on a computer screen by pressing a space bar in response to every letter except the letter ‘X’. Of the various dependent measures, omissions, commissions, hit reaction time, and detectability were the most commonly used indexes and have been used in previous studies [30]. A lower T-score in the CPT indicates better performance, and this score is commonly used in analysis. The Confidence Index (percentile) incorporates all relevant CPT data and is a numerical value up to 100 that specifies the probability that a significant attention problem exists [31].

### 2.4. Statistical Analysis

Data were analyzed using the statistical software package SPSS, version 21.0 (SPSS Inc., Chicago, IL, USA) and variables are presented as either mean (with standard deviation) or frequency. Categorical variables were compared between ADHD patients and healthy controls using either the chi-square test or Fisher’s exact test, depending on the number of cases. Continuous variables were shown as mean (standard deviation), median, and first- and third-quartiles. Mann–Whitney U test was used to determine the differences in BDNF and CNTN1 levels. The Spearman’s correlation was used to analyze the relationships between BDNF and CNTN1 levels, clinical symptoms and neuropsychological functions. Two-tailed *p* values of < 0.05 were considered to indicate statistical significance. We used Bonferroni correction to adjust for multiple testing in the correlation matrix.

## 3. Results

The study sample comprised 136 patients with ADHD (79.4% were boys, mean age: 8.8 years) and 71 healthy controls (77.6% were boys, mean age: 9.6 years). In the sex-stratified analysis (Table 1), the girls with ADHD formed the youngest group, and the healthy control girls were the oldest group. The boys and girls with ADHD had lower scores on all WISC-IV indices than the control boys and girls, respectively. The ADHD group exhibited more severe clinical ADHD symptoms (based on parent-rated and teacher-rated inattention scores, hyperactivity/impulsivity scores, and oppositional SNAP-IV scores) and performed worse in the CPT, median and first (25%) and third quartiles (75%) of demographic data (Table S1).

**Figure 1:**
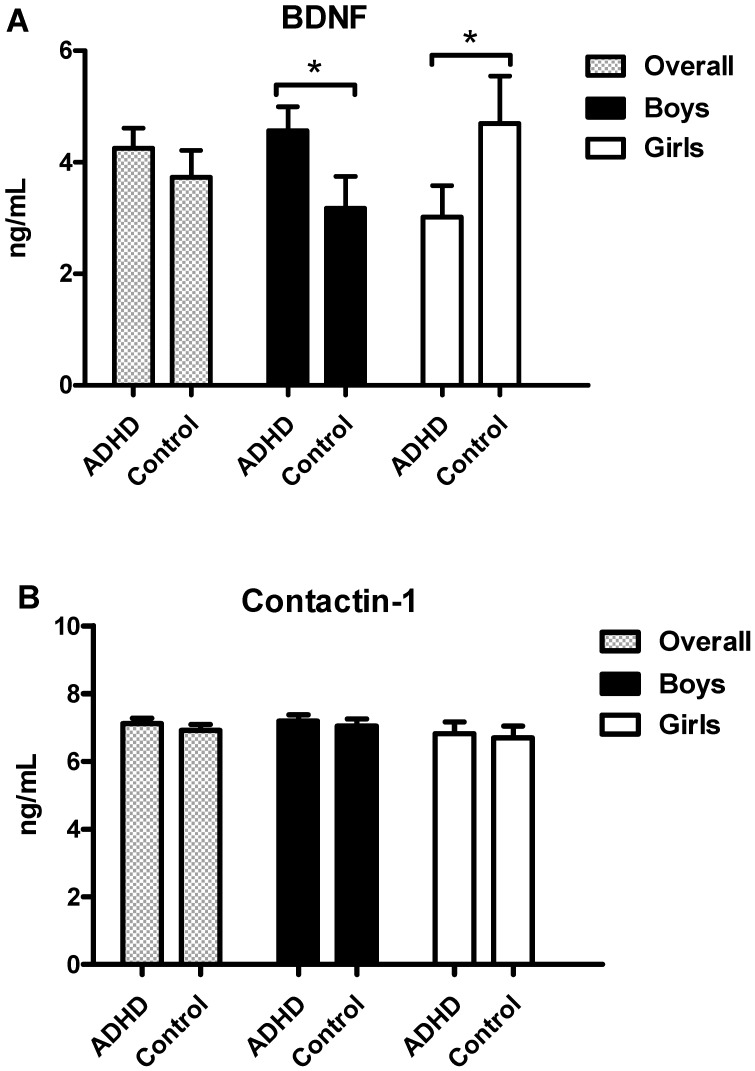
Plasma levels of BDNF (**A**) and contactin-1 (**B**) in patients with ADHD and healthy controls among all subjects, boys and girls. * *p* < 0.05.

The plasma levels of BDNF and CNTN1 in all ADHD patients did not differ significantly from those in the control group (Figure 1). However, the boys with ADHD had higher BDNF levels than the healthy controls boys (Figure 1A) (ADHD: 4.57 ± 4.43 ng/mL vs. Controls: 3.17 ± 3.84 ng/mL, *p* = 0.027, Standardized Test Statistic = 2.210) and the girls with ADHD had lower BDNF levels than the healthy control girls (ADHD: 3.01 ± 2.99 ng/mL vs. Controls: 4.69 ± 4.36 ng/mL, *p* = 0.014, Standardized Test Statistic = 2.458). The gender-stratified analysis revealed no significant differences in CNTN1 levels (Figure 1B) between patients and controls.

Table 2 presents the correlation between BDNF and CNTN1 levels and ADHD clinical symptoms in boys (*N* = 153) and girls (*n* = 54), separately. In boys, BDNF levels were negatively correlated with FSIQ (r = −0.197, *p* = 0.015) and VCI (r = −0.255, *p* = 0.002), and positively correlated with ODD symptoms as rated by parents (r = 0.167, *p* = 0.041) and teachers (r = 0.213, *p* = 0.011). In girls, BDNF levels negatively correlated with inattention symptoms rated by parents (r = −0.322, *p* = 0.019) and omission score in the CPT (r = −0.356, *p* = 0.008). Among both boys and girls, levels of CNTN1 were not significantly related with either ADHD clinical symptoms or neuropsychological functions. However, if we used Bonferroni correction to adjust for multiple testing in the correlation matrix, none of the results maintained their significance.

## 4. Discussion

The main finding in this study is that BDNF levels in ADHD boys exceeded those in control boys, but BDNF levels in ADHD girls were lower than those in control girls. CNTN1 levels did not significantly differ between patients and controls, whether they were boys or girls. BDNF levels were negatively correlated with intelligence in boys, but negatively correlated with the omission errors of girls in the CPT.

Several case-control studies have indicated that the peripheral BDNF levels in both serum and plasma do not differ significantly between patients with ADHD and controls [13,15,16]. A recent meta-analysis revealed no significant difference between peripheral BDNF levels in ADHD patients and control groups [14]. Our findings are therefore consistent with earlier investigations. However, Zhang et al. showed that according to a sex-stratified analysis, BDNF levels are significantly higher in males with ADHD than in controls [14]. The findings herein support that result of Zhang et al. Furthermore, the potential relationship between BDNF, neuropsychological function, and behavioral symptoms was analyzed in the present study. We found that children with greater oppositional defiant symptoms had higher BDNF levels. One possible explanation for the positive correlation between BDNF levels and ADHD severity among boys is that BDNF plays an important role in neuronal growth and plasticity, which is crucial for learning and memory [7]. A previous brain imaging study revealed that ADHD patients that also had comorbid oppositional defiant disorder were related to volumetric reductions in the frontal lobes [32]. Therefore, we hypothesize that BDNF may be compensatorily elevated to facilitate neurodevelopment among boys whose brain development is relatively immature. However, the mechanisms that underlie this role remain undetermined in this study and warrant further investigation.

The data herein reveal that BDNF levels in girls with ADHD were lower than in healthy controls. Among girls, BDNF levels were negatively correlated with omission errors associated with neuropsychological performance and showed a negative correlation between inattention symptoms. This finding demonstrates that BDNF may serve as a protective factor against ADHD among girls whereas the opposite is true for boys. The reasons for the contrary effects of BDNF on the risks of ADHD in boys and girls remain unclear. Sex-related hormonal, genetic, and epigenetic factors interact with BDNF signaling at various levels and in complex ways between genders [18]. The results herein support a sex-specific relationship between peripheral blood BDNF levels and ADHD. Previous epidemiological studies have conclusively established that ADHD is a male-dominant neurodevelopmental disorder (The ratio of boys to girls with this disorder is in the range 2:1 to 9:1) [6]. This sex-specific association is consistent with numerous other studies, which have suggested that many ADHD risk genes had sexually dimorphic effects [33]. Previous investigations have suggested that the gene expression and activity of BDNF may be affected by the different hormonal environments of males and females, which would thus explain the sexually dimorphic effects of BDNF [34].

CNTN1 is involved in the formation of axon connections in the developing nervous system [21], and enhances neuronal excitability through GABAergic neurons [22]. CNTN1, a cell adhesion molecule expressed in developing nervous systems, is vital for the neuronal developmental process formation of newborn cortical neurons, including neural cell adhesion, neurite outgrowth, axon guidance, and myelination [20]. This study is the first to address the relationship between CNTN1 level and ADHD and hypothesize that CNTN1 may serve as a protective factor of ADHD. Nevertheless, we observed no significant relationship between CNTN1 level and ADHD in either boys or girls. This negative finding with regard to CNTN1 may be related to this study’s low statistical power or the lack of the direct relationship between peripheral CNTN1 levels and ADHD phenotypes.

This study has limitations. First, it is a cross-sectional study. Although a relationship was found between peripheral BDNF levels and ADHD, causality cannot be determined. Second, our sample size was small, particularly with respect to females. As a result, the negative findings with regard to the female group may be related to low statistical power. In general, girls with ADHD are diagnosed slightly later than boys [35]. However, in this study, the girls with ADHD had the youngest age among the four groups, which may indicate that the ADHD girls in our study may not be representative of the overall population. Third, the ages of the ADHD and control groups of both boys and girls were not perfectly matched. However, we did not have information about parental education, family socioeconomic status, year in school, and exercise. Although ANCOVA was used to eliminate the confounding effect of age, the results herein may be confounded by the characteristics of patient groups. Fourth, the BDNF and CNTN1 levels were measured in peripheral blood and the peripheral levels do not necessarily reflect the levels in CNS. Moreover, the polymorphisms of BDNF and CNTN1 genes may influence the effects of BDNF and CNTN1 levels. For example, several studies have implicated BDNF as a genetic risk factor for ADHD [36,37]. A sex-specific relationship between the BDNF Val66Met genotype and plasma BDNF levels has also been observed in a Han-Chinese sample [38]. However, we did not identify the genotypes of BDNF and CNTN1 genes in this study. Finally, if we adjusted for multiple testing in the correlation matrix with Bonferroni correction or if only ADHD patients were considered in the analyses, none of the significant results remained. The robustness of the relationships between BDNF levels and ADHD characteristics needs to be further investigated.

## 5. Conclusions

The results herein suggest that BDNF may be involved in sex-specific susceptibility to ADHD. However, CNTN1 was not associated with ADHD pathophysiology. Whether peripheral BDNF levels serve as a potential biomarker for ADHD warrants further investigation in the future. A longitudinal study with a larger sample or an experimental study is required to elucidate the mechanism of the sex-specific relationship between BDNF and ADHD.

## Figures and Tables

**Table 1:** Comparisons of demographic data and psychopathology evaluations in boys and girls among patients with ADHD and healthy controls.

	Boys		Girls	
	ADHD (*n* = 108)	Control (*n* = 45)	ADHD (*n* = 28)	Control (*n* = 26)
Demographic data, mean (SD)				
Age, years	9.0 (2.3)	9.4 (2.4)	8.0 (1.3)	9.9 (2.6)
Height, cm	135.1 (14.9)	136.8 (14.7)	127.8 (10.5)	139.5 (14.9)
Body weight, kg	35.0 (13.7)	34.2 (11.2)	30.0 (12.0)	35.7 (11.9)
Body Mass Index	18.5 (4.0)	17.7 (2.8)	17.8 (4.5)	17.8 (3.1)
Comorbidities, *n* (%)				
ODD or conduct disorder	28 (26.2)	-	4 (14.3)	-
Tic disorders	16 (15.0)	-	1 (3.6)	-
WISC-IV, mean (SD)				
Full Scale Intelligence Quotient	98.1 (10.9)	109.3 (14.7)	98.0 (9.0)	105.1 (11.2)
Verbal Comprehension Index	101.2 (11.7)	108.1 (12.7)	102.6 (8.6)	103.3 (12.1)
Perceptual Reasoning Index	99.6 (12.7)	110.7 (17.0)	95.9 (10.1)	106.2 (14.0)
Working Memory Index	99.5 (12.1)	108.7 (12.6)	99.3 (9.6)	105.1 (11.9)
Processing Speed Index	93.5 (9.7)	100.6 (11.9)	95.3 (8.3)	101.9 (11.1)
SNAP-IV, mean (SD)				
SNAP-IV parent form (I)	16.4 (5.7)	6.4 (6.2)	16.6 (4.8)	4.9 (4.5)
SNAP-IV parent form (H)	14.9 (6.7)	5.4 (5.6)	13.2 (5.1)	3.5 (5.7)
SNAP-IV parent form (O)	12.2 (6.1)	5.8 (5.4)	11.0 (5.9)	4.7 (5.4)
SNAP-IV teacher form (I)	15.2 (5.4)	4.8 (5.0)	13.9 (7.3)	4.0 (3.8)
SNAP-IV teacher form (H)	13.0 (6.8)	3.4 (3.5)	7.8 (6.1)	1.8 (2.3)
SNAP-IV teacher form (O)	9.5 (6.2)	2.2 (3.5)	5.0 (4.5)	1.4 (1.7)
Conners’ CPT, mean (SD)				
Confidence Index	64.5 (22.8)	54.8 (18.9)	60.9 (23.7)	37.3 (18.2)
Omission	59.9 (21.0)	53.1 (16.7)	62.1 (14.5)	49.4 (6.9)
Commission	49.5 (9.9)	43.7 (11.3)	46.5 (7.9)	48.8 (8.6)
Hit Reaction Time	55.5 (14.5)	57.9 (11.4)	59.2 (7.9)	54.2 (10.5)
Detectability	51.8 (8.8)	46.1 (12.1)	48.2 (8.7)	50.2 (8.1)

Notes: Data are expressed as mean (SD) or *n* (%); H/I, hyperactive/impulsive type; ODD, oppositional defiant disorder; SNAP-IV, the Swanson, Nolan, and Pelham–Version IV Scale for ADHD; WISC-IV, the Wechsler Intelligence Scale for Children–Fourth Edition; CPT, Conners’ Continuous Performance Test; I, inattention scores; H, hyperactivity/impulsivity scores; O, oppositional scores.

**Table 2:** Correlation among BDNF and contactin-1 and clinical assessments in boys and girls among patients with ADHD and healthy controls, respectively ^a^.

Variables	BDNF				Contactin-1			
	Boys		Girls		Boys		Girls	
	r	*p*-Value	r	*p*-Value	r	*p*-Value	r	*p*-Value
WISC-IV								
Full Scale Intelligence Quotient	−0.197	0.015 *	0.004	0.978	−0.027	0.741	0.124	0.376
Verbal Comprehension Index	−0.255	0.002 *	−0.053	0.707	−0.069	0.400	−0.008	0.955
Perceptual Reasoning Index	−0.117	0.155	0.093	0.514	−0.078	0.344	0.098	0.489
Working Memory Index	−0.127	0.122	−0.075	0.593	−0.044	0.591	0.137	0.327
Processing Speed Index	−0.107	0.192	−0.036	0.800	0.018	0.829	0.134	0.339
SNAP-IV								
SNAP-IV parent form (I)	0.080	0.332	−0.322	0.019*	−0.063	0.444	−0.022	0.873
SNAP-IV parent form (H)	0.116	0.156	−0.233	0.099	−0.022	0.792	−0.050	0.721
SNAP-IV parent form (O)	0.167	0.041 *	−0.175	0.211	0.057	0.486	−0.019	0.892
SNAP-IV teacher form (I)	0.094	0.265	−0.168	0.234	−0.057	0.501	−0.175	0.215
SNAP-IV teacher form (H)	0.128	0.127	−0.145	0.305	0.042	0.622	0.060	0.673
SNAP-IV teacher form (O)	0.213	0.011*	−0.085	0.548	0.135	0.108	−0.097	0.496
Conners’ CPT								
Confidence Index	−0.010	0.899	−0.185	0.180	−0.026	0.752	0.069	0.618
Omission	−0.069	0.401	−0.356	0.008*	−0.085	0.299	−0.064	0.647
Commission	0.077	0.351	0.167	0.229	0.073	0.377	−0.075	0.591
Hit Reaction Time	−0.119	0.147	−0.083	0.551	−0.108	0.190	−0.074	0.593
Detectability	0.097	0.149	0.144	0.298	−0.054	0.513	0.137	0.325

^a^ Data are expressed by Spearman’s correlation coefficient. SNAP-IV, the Swanson, Nolan, and Pelham–Version IV Scale for ADHD; WISC-IV, the Wechsler Intelligence Scale for Children–Fourth Edition; I, inattention scores; H, hyperactivity/impulsivity scores; O, oppositional scores. * *p* < 0.05.

## Supplementary Materials

The following are available online at https://www.mdpi.com/2077-0383/8/9/1366/s1, Table S1. Median and first (25%) and third quartiles (75%) of demographic data and psychopathology evaluations in boys and girls among patients with ADHD and healthy controls.
