# Supplementary material for: Peripheral Brain-Derived Neurotrophic Factor and Contactin-1 Levels in Patients with Attention-Deficit/Hyperactivity Disorder
Source: J Clin Med. 2019 Sep 2;8(9):1366. doi: 10.3390/jcm8091366 (PMC6780884; doi:10.3390/jcm8091366)
Supplement: Supplementary file 1 [file jcm-08-01366-s001.pdf]

Supplementary Table 1 Median and first (25%) and third quartiles (75%) of demographic data and psychopathology evaluations in boys and girls among patients with ADHD and healthy controls.

	Boy						Girl					
	ADHD			Control			ADHD			Control		
	25%	Median	75%	25%	Median	75%	25%	Median	75%	25%	Median	75%
Demographic data												
Age, years	7.2	8.6	11.0	7.5	9.3	10.9	6.8	7.8	9.0	7.8	9.8	11.5
Height, cm	125.0	132.0	146.0	125.0	136.0	144.0	120.5	127.0	130.0	126.5	144.0	153.0
Body weight, kg	24.0	30.4	44.0	25.0	32.0	40.0	23.0	25.0	31.0	23.5	37.0	48.0
WISC-IV												
Full Scale Intelligence Quotient	92.0	99.0	107.0	100.0	107.0	118.0	92.0	100.0	102.5	94.5	104.0	113.5
Verbal Comprehension Index	95.0	101.0	107.0	99.0	107.0	115.0	95.0	105.0	109.0	94.0	101.0	115.0
Perceptual Reasoning Index	91.0	100.0	108.0	97.0	105.0	126.0	91.0	95.0	100.0	95.0	103.0	116.5
Working Memory Index	94.0	100.0	108.0	103.0	108.0	116.0	94.0	100.0	104.5	94.0	106.0	114.0
Processing Speed Index	86.0	97.0	99.0	97.0	99.0	108.0	92.0	94.0	99.0	94.0	99.0	106.5
SNAP-IV												
SNAP-IV parent form (I)	12.0	16.0	20.0	1.0	4.0	9.0	12.5	16.0	20.5	1.0	4.0	6.5
SNAP-IV parent form (H)	10.0	14.0	20.0	0	3.0	7.0	9.0	12.0	16.5	0	1.0	3.5
SNAP-IV parent form (O)	8.0	12.0	16.0	1.0	3.0	8.0	5.5	11.0	15.5	1.0	3.0	6.0
SNAP-IV teacher form (I)	11.0	16.0	19.0	1.0	3.0	6.0	6.5	12.0	19.5	0	3.0	6.0
SNAP-IV teacher form (H)	8.0	13.0	18.0	0	2.0	5.0	2.0	6.0	12.0	0	0	2.5
SNAP-IV teacher form (O)	4.0	9.0	14.0	0	1.0	2.0	1.0	3.0	7.5	0	0	2.5
Conners' CPT, mean ± SD												
Confidence Index	48.4	56.0	87.1	41.9	50.0	67.4	39.9	63.6	79.8	25.3	44.4	50.0
Omission	43.8	51.2	70.0	43.8	47.7	56.3	51.9	58.4	71.6	44.0	48.8	53.3
Commission	43.8	49.8	57.1	38.4	46.4	51.1	39.5	48.1	52.4	42.3	51.0	56.7
Hit Reaction Time	45.7	54.0	62.1	49.2	58.7	66.2	54.7	58.6	65.0	45.4	55.5	62.3
Detectability	47.0	52.4	58.1	39.8	48.9	54.8	41.3	46.8	54.4	44.3	49.6	57.5

Notes: H/I, hyperactive/impulsive type; ODD, oppositional defiant disorder; SNAP-IV, the Swanson, Nolan, and Pelham–Version IV Scale for ADHD; WISC-IV, the Wechsler Intelligence Scale for Children–Fourth Edition; CPT, Conners' Continuous Performance Test; I, inattention scores; H, hyperactivity/impulsivity scores; O, oppositional scores.
